# Assessment of Tryptophan, Tryptophan Ethylester, and Melatonin Derivatives in Red Wine by SPE-HPLC-FL and SPE-HPLC-MS Methods

**DOI:** 10.3390/foods8030099

**Published:** 2019-03-14

**Authors:** Daniela Fracassetti, Ileana Vigentini, Alfredo Fabrizio Francesco Lo Faro, Patrizia De Nisi, Roberto Foschino, Antonio Tirelli, Marica Orioli, Marcello Iriti

**Affiliations:** 1 Department of Food, Environmental and Nutritional Sciences, Università degli Studi di Milano, Via G. Celoria 2, 20133 Milan, Italy; ileana.vigentini@unimi.it (I.V.); roberto.foschino@unimi.it (R.F.); antonio.tirelli@unimi.it (A.T.); 2 Department of Biomedical, Surgical and Dental Sciences, Università degli Studi di Milano, Via Mangiagalli 37, 20133 Milan, Italy; fabriziolofaro09@gmail.com (A.F.F.L.F.); marica.orioli@unimi.it (M.O.); 3 Department of Agricultural and Environmental Sciences - Production, Landscape, Agroenergy, Università degli Studi di Milano, Via G. Celoria 2, 20133 Milan, Italy; patrizia.denisi@unimi.it (P.D.N.); marcello.iriti@unimi.it (M.I.)

**Keywords:** indoleamines, grapevine, liquid chromatography, UHPLC/ESI-QTRAP, wine

## Abstract

Melatonin (MEL) is an indoleamine produced mainly by the pineal gland in vertebrates. It plays a significant role in the regulation of circadian rhythms, mitigation of sleeping disorders, and jet lag. This compound is synthetized from tryptophan (TRP) and it has been found in seeds, fruits, and fermented beverages, including wine. Wine is also a source of other tryptophan derivatives, the tryptophan ethylester (TEE) and MEL isomers (MISs), for which the biological properties need to be elucidated. An analytical method for the simultaneous quantification of TRP, TEE, and MEL was developed by a Solid Phase Extraction (SPE) of a preconcentration of wine followed by high performance liquid chromatography (HPLC) analysis either with fluorescence or mass spectrometer detectors. The analytical method showed a relative standard deviation (RSD) lower than 8%, except for TRP (RSD 10.5% in wine). The recovery was higher than 76%. The versatility of SPE preconcentrations allowed for the adequate preconcentration of wine sample as well as detection of low concentrations, an important aspect especially for MEL (detection limit 0.0023 µg/L). The proposed method proved to be suitable for assessing the investigated compounds in some red wine samples, where 74.4–256.2 µg/L and 0.038–0.063 µg/L of TEE and MEL were detected, respectively. Five MISs were also found in wine samples in concentrations up to 1.97 µg/L.

## 1. Introduction

Melatonin (*N*-acetyl-5-methoxytryptamine; MEL) is an indoleamine produced mainly by the pineal gland in vertebrates [1] and its synthesis also occurs in other tissues, such as the gastrointestinal tract, bone marrow, and lymphocytes [2,3,4]. It is synthetized from l-tryptophan metabolism via serotonin [5,6]. In animals, MEL modulates the circadian and circannual rhythms, reproductive functions, bone metabolism, and turnover via cell-receptor-mediated mechanisms. It also shows a powerful antioxidant activity by directly scavenging free radical species (both reactive oxygen and nitrogen species) and stimulating the activity of antioxidant enzymes [7]. MEL has been found in edible plants [8,9,10] as a phytohormone protecting against oxidative stress and regulating growth [11,12]. Consequently, MEL is also present in plant foods, including seeds, fruits, and fermented beverages [11,13,14]. Several authors evidenced the presence of MEL in wines [15,16,17,18]. The circulating levels of MEL in mammals are very low (about 200 pg/mL at the maximum night peak and lower than 10 pg/mL during the day) [19] compared to MEL in grape products (about 1000 pg/g in berry skin and 500 pg/mL in wine) and, therefore, the intake of grape products represents a relevant topic [16,20]. The content of MEL increases during the fermentation step of the winemaking and other fermented beverages, meaning the yeast plays a significant role in its biosynthesis [14,21,22,23]. Besides MEL, its isomers (MISs) were also detected, and one of them was recently identified as tryptophan-ethyl ester (TEE) [24,25]. This compound was the most abundant tryptophan derivative, and its concentration was higher than that of MEL [26,27]. 

The analytical methods described for the detection of MEL in wine were based on liquid chromatography separation coupled with fluorescence [15,28,29] and mass spectrometry detectors [21,26,27]. Recently, Muñiz-Calvo and coauthors [30] proposed voltammetric techniques for the determination of MEL, TRP, and other TRP derivatives (TEE excluded). However, the authors evidenced the high performance liquid chromatography (HPLC) analysis had high discriminating capability and unambiguous identification and quantification. Due to the fact that wine is a complex matrix and MEL and its derivatives are present in low concentrations, the use of a solid phase extraction (SPE) technique is beneficial to use due to its removal of interferences, isolation, and concentration capabilities.

This study aimed to develop an HPLC method for the simultaneous determination of TRP, MEL, and TEE. In this respect, we developed a sample preparation protocol by means of SPE preconcentration allowing the detection of the three compounds by fluorescence and mass spectrometry detectors. The proposed method was used to determine the content of TRP, MEL, MISs, and TEE in red wine samples.

## 2. Materials and Methods 

### 2.1. Chemicals and Materials

Melatonin (MEL), tryptophan-ethyl ester (TEE), methanol, and formic acid were purchased from Sigma-Aldrich (St. Louis, MO, USA). All the chemicals were of analytical grade. HPLC grade water was obtained by a Milli-Q system (Millipore Corp., Bedford, MA, USA). 

MEL and TEE stock solutions were prepared in an ethanol:water ratio of 80:20 at 100 mg/L, while TRP was dissolved in water at 1000 mg/L. The stock solutions were stored covered with aluminum foil in order to protect the compounds against the light at −20 °C and they were thawed once. 

Synthetic wine solution (SWS) contained 5.0 g/L tartaric acid and 12% ethanol (*v*/*v*) at a pH of 3.2, adjusted with sodium hydroxide (Merck, Darmstadt, Germany).

Eight red wine samples were analyzed. The wines were produced from *Vitis vinifera* cv. Nebbiolo in vintage 2015 in the Valtellina area (Lombardia, Italy), and they were collected at the market.

### 2.2. Sample Preparation

The preconcentration of red wine was developed by means of a solid phase extraction (SPE) technique. The SPE cartridges tested were Strata C18-T 500 mg/3 mL and Strata X-Polymeric Reversed Phase 200 mg/3 mL (Phenomenex, Torrance, CA, USA). 

The final SPE protocol was set with Strata X-Polymeric Reversed Phase 200 mg/3 mL. After the SPE activation with methanol (4 mL) and conditioning with 0.1% formic acid (*v*/*v*) (8 mL), 5 mL of sample was loaded and the eluate was recovered (fraction A). The cartridge was firstly washed with 5 mL 0.1% formic acid in water (*v*/*v*) (fraction B) and then with 5 mL of 40% methanol (*v*/*v*) (fraction C). The elution was carried out with 5 mL of 100% methanol (fraction D), which was evaporated under vacuum (Rotavapor R 110, Büchi). The sample was re-suspended in 500 µL of 10% methanol acidified with 0.1% formic acid (*v*/*v*) corresponding to a concentration fold of 10. Each fraction was analyzed after filtration through a 0.22 μm polyvinilidene fluoride (PVDF) filter (Millipore). Samples were protected from the light during the preparation. 

The setting of the SPE procedure was carried out on both spiked SWS and red wine at two concentration levels: 1 mg/L TRP, 50 μg/L TEE, and 50 μg/L MEL (level 1); and 2 mg/L TRP, 500 μg/L TEE, and 500 μg/L MEL (level 2). A higher concentration of TRP was chosen because its average content has been reported to be 3 mg/L [31]. The SPE procedure setup included differences in (i) volume of sample loaded (5 mL and 10 mL); (ii) volume of washing with 0.1% formic acid (*v*/*v*) (5 mL and 10 mL); (iii) volume and composition of washing with methanolic solution (5 mL and 10 mL; methanol 40%, 50%, 60%, and 70% (*v*/*v*)); (iv) recovery of fraction D with a different methanolic solution (methanol 50% (*v*/*v*), methanol 50% (*v*/*v*) acidified with 0.1% formic acid (*v*/*v*), 10% methanol (*v*/*v*), and 10% methanol (*v*/*v*)acidified with 0.1% formic acid (*v*/*v*)). 

### 2.3. Chromatographic and Quantification Conditions

The HPLC system consisted of an Agilent 1260 Infinity Quaternary LC equipped with an Agilent G1321B Fluorescence Detector and an Agilent LC/MS 6130 Quadrupole (Agilent, Santa Clara, CA, USA) operating in multimode source (simultaneous electrospray (ESI) and atmospheric pressure chemical ionization (APCI)). The MS operative conditions included a spray voltage of +3.5 kV, vaporizer temperature of 250 °C, gas temperature of 350 °C, drying gas at 12 mL/min, and a nebulizer pressure of 55 psig. The fluorescence detector was set at 280 nm and 350 nm for excitation and emission, respectively. The column used for the separation was an Accucore C18 (100 × 3 mm, 2.7 µm particle size, Thermo Scientific, San Jose, CA, USA) set at 40 °C. The elution solvents were: (A) 0.1% formic acid (*v*/*v*), and (B) methanol acidified with 0.1% formic acid (*v*/*v*), and the flow rate was 0.5 mL/min. Elution conditions were as follows: 5% B for 1 min, from 5% to 40% B in 15 min followed by the column washing with 100% B and re-equilibration for a total of 25 min for each run. Injection volumes were 5 µL and 20 µL for HPLC-MS and HPLC-FL methods, respectively. 

Chromatographic data were processed using Agilent OpenLab ChemStation software (Agilent). For the MS detector, data acquisition was performed in a single ion monitoring (SIM) setting [m/z]+ 205 for TRP and 233 [m/z]+ for MEL and TEE. MEL, TEE, and TRP were quantified using the external standard method. Six-level calibration curves were obtained with standard solutions containing the analytes at the respective concentrations spanning the expected ranges in wine. 

The UHPLC/ESI-QTRAP analysis was carried out to identify MEL, MISs, and TEE eluting in the chromatograms of the wine samples. A UHPLC model HP 1290 (Agilent) coupled with a Mass Spectrometer Detector 5500 TRIPLE QTrap model 1024945-AX (ABSCIEX, Framingham, MA, USA) equipped with an HESI-II probe for electrospray ionization and a collision cell (HCD) was used. Data were acquired in a multiple reaction monitoring (MRM) mode. The operative conditions were: spray voltage +2.2 kV, sheath gas flow-rate 50, auxiliary gas flow-rate 55, capillary temperature 360 °C, capillary +95 V, tube lens +170 V, Skimmer +38 V, and heater temperature 500 °C. The chromatographic separation was carried out on a BEH C18 column (100 × 2.1 mm, 1.7 µm particle size, Waters, Milford, MA, USA). The column was maintained at 30 °C and the injection volume was 0.3 μL. The elution solvents were (A) 0.1% formic acid (*v*/*v*), and (B) acetonitrile. The elution conditions were as follows: 10% B for 0.30 min, from 10% to 30% B in 5 min, from 30% to 85% in 0.5 min followed by column washing with 90% B and re-equilibration for a total of 10.5 min for each run. The flow rate was set at 0.55 mL/min. The MS data were processed using MultiQuant software (AB Sciex). The peak identity was ascertained by evaluation of both the accurate mass and the fragments obtained in the collision cell. MEL, TEE, and TRP were quantified using the external standard method with the respective standards; MISs were quantified with the calibration curve obtained for MEL.

### 2.4. Method Validation

The in-house validation of the method was carried out in terms of selectivity, linearity, limit of detection (LOD), limit of quantification (LOQ), repeatability, and recovery [32]. 

The selectivity of the method was evaluated by analyzing TRP, TEE, and MEL both in the absence and presence of possible interferences originating from the wine matrix. With this aim, the separations of a SWS at the concentrations 2 mg/L TRP, 50 μg/L TEE, and 50 μg/L MEL analyzed with both FL and MS detectors were compared with those of a purified SWS and a red wine sample spiked with the three analytes at the same concentrations.

Linearity was tested on six concentration levels within the intervals reported in Table 1 and Table 2. The SWS samples were purified and analyzed in triplicate as well as spiked red wine samples at the same concentration levels. The equations of the calibration curves and the correlation coefficients (*r*) were obtained by linear regression analysis. 

The values of limit of detection (LOD) and limit of quantification (LOQ) were calculated as the lowest concentration of analyte in a sample that resulted in a signal-to-noise ratio of 3 and 10 for LOD and LOQ, respectively. Values were measured in three independent replicates.

The recovery (%) in purified SWSs and spiked wine samples was calculated using the SWSs added with the same concentrations of the analytes. The spiking concentrations were 5.3 µg/L, 10.6 µg/L, 21.2 µg/L, 53.0 µg/L, 106.0 µg/L, and 212 µg/L for TEE and MEL, and 110 µg/L, 220 µg/L, 550 µg/L, 1100 µg/L, 2200 µg/L, and 5500 µg/L for TRP. SWSs and spiked red wine samples were purified and analyzed in triplicate. Triplicate analyses of unpurified SWSs spiked with the same concentrations of the analytes were also carried out. The accuracy was evaluated by means of recovery assay of replicate preconcentrations of both SWS and spiked red wine samples at levels of 10.6 µg/L and 106.0 µg/L for TEE and MEL, and 220 µg/L and 2200 µg/L for TRP. Duplicate preconcentrations were carried out in two different days for SWSs (*n* = 4) and for three different days for spiked red wine samples (*n* = 6). 

Precision was expressed as relative standard deviation (RSD) of the analytical response. Both the SWSs and red wine samples spiked at the three levels of concentration were analyzed (10.6 µg/L, 53.0 µg/L, and 106.0 µg/L for TEE and MEL, and 220 µg/L 1100 µg/L, and 2200 µg/L for TRP). Three independent replicates were carried out on three different days (*n* = 9). For the repeatability of the SPE procedure and instrumental repeatability, duplicate injections were carried out from the same vial at the two concentration levels on three different days (*n* = 12). The repeatability of the retention times was also evaluated over a total of 54 injections carried out on three different days.

### 2.5. Statistical Analysis

Statistical analysis was carried out with SPSS Win 12.0 program (SPSS Inc., Chicago, IL, USA). The equations of the calibration curves were assessed by the linear regression analysis. Differences between the calibration curve slopes obtained in aqueous solution and white wine were evaluated by the F-test (*p* < 0.05).

## 3. Results and Discussion

### 3.1. Analytical Method Development

**Figure 1:**
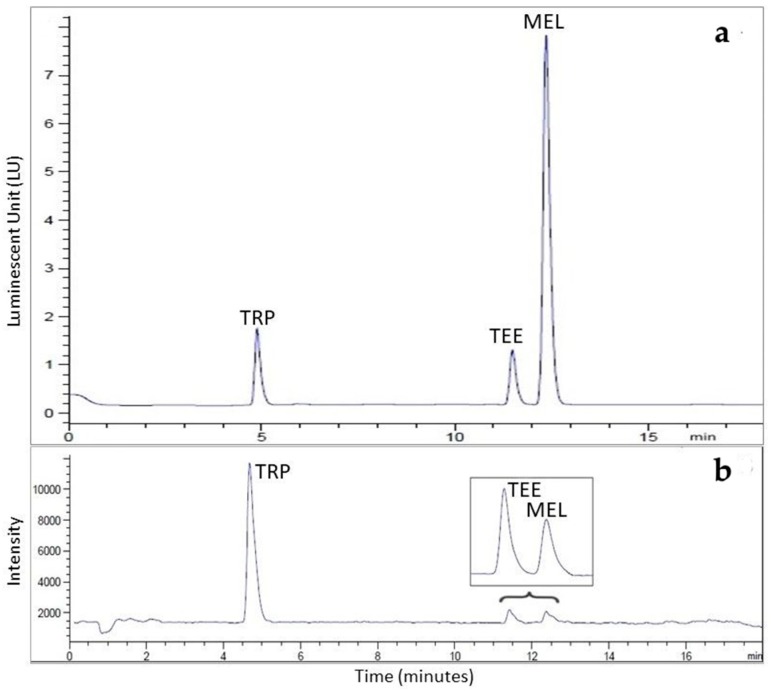
(**a**) HPLC-FL and (**b**) HPLC-MS chromatograms of tryptophan (TRP) (1 mg/L), tryptophan ethyl ester (TEE) (50 μg/L), and melatonin (MEL) (50 μg/L) in standard synthetic wine solution.

The chromatographic conditions by means of both FL and MS detectors were firstly optimized using the SWS at the concentration 2 mg/L TRP, 50 μg/L TEE, and 50 μg/L MEL. While the analytical response (area) was similar for MEL and TEE using MS detector, it resulted lower in fluorescence for TEE analyzed at the same concentration (Figure 1). 

A standard water solution with the same levels of analytes was analyzed, and no differences were observed in comparison to SWS. The SPE preconcentration allowed both the removal of compounds potentially interfering in the HPLC separation from the wine matrix and the concentration of the analytes of interest, making their detection possible because of their presence in low amounts in wine [33]. Although other authors applied the SPE preconcentration for MEL detection from wine and other food matrices [15,27,29,34], the SPE approach presented in this study allowed the simultaneous elution of TRP, TEE, and MEL. For the development of the SPE preconcentration, several assays were carried out using the SWS containing concentrations of 2 mg/L TRP, 50 μg/L TEE, and 50 μg/L MEL. The setup method by using a blank matrix added with the compounds of interest is considered acceptable [35] since a Certified Reference Material for wine containing TRP, TEE, and MEL does not exist. First, preconcentrations by using two different SPE cartridges were compared by loading 10 mL of spiked SWS and washing with methanol 40% methanol (*v*/*v*). Higher recovery (>15%) was observed by the preconcentration with Strata X-Polymeric Reversed Phase 200 mg, in accordance to El-Moussaoui and Bendriss [29]. These SPE cartridges were used for the development of the analytical method. Trials loading 5 mL and 10 mL of spiked SWS at two concentration levels (see Paragraph 2.2), and washing with 5 mL and 10 mL 0.1% formic acid (*v*/*v*), and 5 mL 40% methanol (*v*/*v*), led to comparable recovery values. The lower amounts were chosen in order to reduce the solvent amounts needed for the SPE, which were 5 mL of 0.1% formic acid and 5 mL of methanol in total for the four steps of preconcentration. The analyses of all these fractions were carried out. TRP was detected in the sample loading (fraction A) and in the washing with 40% methanol (fraction C), TEE was revealed in fraction C and in the methanolic solution (fraction D), while MEL was only found in fraction D. The major interferences of red wine are represented by phenols, compounds completely soluble in methanol. In order to reduce the possible interferences in fraction D, different concentrations of methanol (40–70% (*v*/*v*)) were assayed. However, the increase of methanol in the washing step led to a loss of MEL in fraction D, even when the washing was carried out with 50% methanol (*v*/*v*). The recovery of fraction D, after methanol evaporation under vacuum, was carried out with different methanol amounts (500 µL and 1 mL) and concentrations (10% and 50% (*v*/*v*)), with and without acidification with 0.1% formic acid (*v*/*v*). The best chromatographic separation in terms of peak shape was obtained with methanol 10% acidified with 0.1% formic acid (*v*/*v*). Formic acid 0.1% (*v*/*v*) was the solvent for the HPLC separation and no change of sample pH was generated through the analysis. 

Based on the results obtained for the spiked SWS, the SPE procedure was carried out by loading 5 mL of spiked SWS and spiked wine sample recovering the eluate (fraction A). The cartridge was firstly washed with 5 mL of 0.1% formic acid (*v*/*v*) (fraction B) and then with 5 mL of 40% methanol (*v*/*v*) (fraction C). The elution was carried out with methanol 100% (fraction D) which was evaporated under vacuum. The fraction D was re-suspended in 500 µL of 10% methanol acidified with 0.1% formic acid (*v*/*v*), meaning a 10-fold concentration of wine sample. 

**Figure 2:**
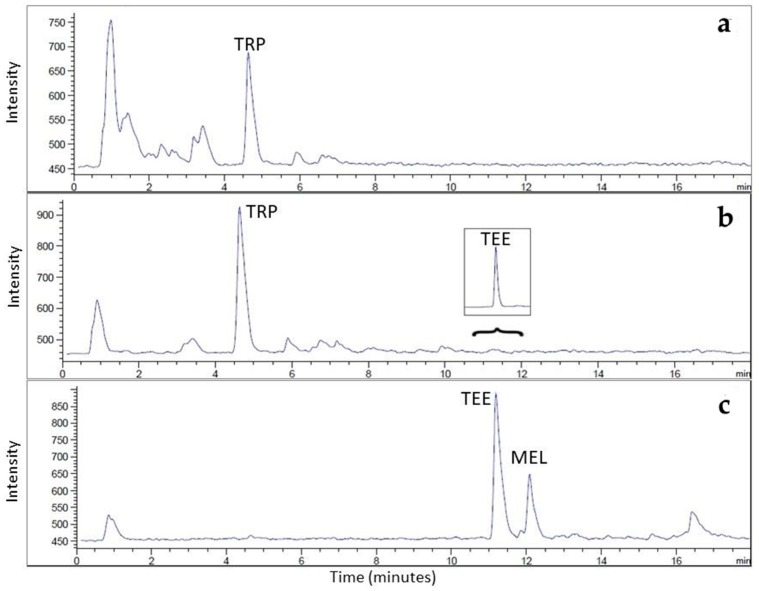
HPLC-MS chromatograms of (**a**) fraction A, (**b**) fraction C, and (**c**) fraction D obtained for solid phase extraction (SPE)-preconcentrated red wine sample spiked with TRP (1 mg/L), TEE (50 μg/L), and MEL (50 μg/L). Peak identity: TRP, tryptophan; TEE, tryptophan ethyl ester; MEL, melatonin.

Before proceeding to method validation, spiked red wine samples at two concentration levels (see Paragraph 2.2) were SPE preconcentrated in order exclude any possible interferences arising from the HPLC separation of red wine, especially for the concentrated fraction D. As shown in Figure 2, the HPLC separation was interference-free, not only for fraction D, but also for fractions A and C.

### 3.2. Validation of the Analytical Method

The linearity was firstly assessed for TRP, TEE, and MEL added to SWS by calculating six-point calibration curves without SPE preconcentrations. Both the FL and MS detectors showed correlation coefficient (r) values higher than 0.99 (Table 1). A linear response was also found for TRP, TEE, and MEL determined in spiked SWSs after SPE preconcentration and analyzed by the FL detector (*y* = 39.03 × *x* + 0.07 for TRP, *y* = 0.51 × *x* + 58.46 for TEE, and *y* = 1.19 × *x* − 9.00 for MEL) with *r* values that corresponded to 1, 0.991, and 0.998 for TRP, TEE, and MEL, respectively. The LOD and LOQ were assessed for the three compounds and they resulted in lower than the average concentrations found in wine [21,28,33,36] for both TRP (5.16 µg/L and 17.18 µg/L for LOD and LOQ, respectively) and 22 MEL (0.12 µg/L and 0.41 µg/L for LOD and LOQ, respectively). Higher LOD and LOQ were found for TEE (1.17 µg/L and 3.91 µg/L, respectively) making the analytical method proposed less sensitive for TEE quantification in comparison to MEL. However, Vigentini et al. [26] and Fernández-Cruz et al. [27] reported concentrations of TEE higher than the LOQ value; the mentioned authors found TEE amounts in most cases higher than 5 µg/L and up to 1126 µg/L. 

A linear response was observed for both spiked SWSs and red wine samples analyzed by the MS detector, and no significant difference was found between the two matrices. The r values were higher than 0.99 for all the compounds investigated, and no significant differences were found in the calibration curve slopes calculated in spiked SWSs and red wine samples purified with SPE (Table 2). As expected, both LOD and LOQ values were lower when the detection was carried out by the MS detector (Table 2), which is more sensitive than FL detector. The LOD values we found were on the same order of magnitude as other analytical methods previously proposed for TEE, while the results were lower in the case of MEL, indicating a high sensitivity of the analytical method proposed [26,27,28,37]. In addition, the chromatographic separation was interference-free (Figure 2), facilitating the identification and quantification. 

The recovery of the SPE preconcentration was evaluated for both spiked SWSs and red wine samples at six concentration levels (see Paragraph 2.4). The average values of percent recovery were all higher than 75% (Table 2). Only negligible differences were obtained by analyzing the spiked SWSs and red wine samples, thus a matrix effect could be excluded (Table 2). 

The repeatability of the method was calculated as the relative standard deviation (RSD) of the analytical response (peak area) obtained by analyzing both spiked SWSs and red wine samples (*n* = 9). The average RSD values obtained for the TRP, TEE, and MEL in spiked SWSs ranged between 4.6% and 9.1%, whereas values obtained for spiked red wine samples were between 5.4% and 10.5% (Table 2). The repeatability of the SPE preconcentration was also evaluated for two concentration levels in both spiked SWS (*n* = 4) and red wine (*n* = 6). The average values determined for spiked SWS were 5.4%, 7.4%, and 4.0%for TRP, TEE, and MEL, respectively, and the values for spiked red wine samples were 4.2%, 5.2%, and 5.2% for TRP, TEE, and MEL, respectively. The intra-day precision (*n* = 12) corresponded to 3.8%, 2.6%, and 3.1% for TRP, TEE, and MEL, respectively. The repeatability of retention times (*n* = 54), estimated on three different days, was negligible (<2%). Overall, these data confirm the reliability of the analytical method here proposed.

The last step of the developed sample preparation method was the evaporation of methanol under vacuum and the re-suspension of samples in 500 µL of 10% methanol acidified with 0.1% formic acid (*v*/*v*), meaning a 10-fold concentration of wine sample. Assays were carried out by concentrating the wine samples up to 25-fold, and results were in accordance to those found for 10-fold concentrated samples (data not shown). These findings indicated the high versatility of the developed SPE preconcentration, making possible the detection of the investigated compounds, MEL and TEE in particular, even when they are present in very low concentrations (<0.02 µg/L).

### 3.3. Analysis of Red Wine Samples

In order to evaluate the suitability of the analytical method proposed, the determinations of TRP, TEE, and MEL were carried out in eight red wine samples produced from *Vitis vinifera* cv. Nebbiolo in vintage 2015. The wine samples were also analyzed by UHPLC/ESI-QTRAP in order to confirm the presence of MEL and TEE. Besides the latter two compounds, five MISs were detected, and fragmentation is reported in Table 3. This is an improvement of the existing methods since one MIs [38,39] to three MISs [26,33] were previously detected in wine. 

The concentration of TRP varied from 0.44 ± 0.05 mg/L to 4.39 ± 0.46 mg/L (Table 4); the concentration of free amino acids can be quite variable in wine, depending on the initial content in grapes and cellular lysis occurrence of wine-related microorganisms [31]. The revealed TEE was variable among wine samples, ranging from 74.4 ± 5.9 µg/L to 256.2 ± 20.2 µg/L (Table 4). These amounts were higher in comparison to the values reported by Vigentini et al. [26] and Fernández-Cruz et al. [27] who monitored the TEE content in laboratory-scale fermentations in real must and in synthetic must, respectively. Maybe winemaking at an industrial-scale or the unknown fermenting yeast could affect the level of TEE. MEL was detected in the analyzed red wine samples, ranging from 0.038 ± 0.001 µg/L to 0.063 ± 0.004 µg/L (Table 4), on the same order of magnitude as the concentration reported by Vitalini and co-authors [33] who detected MEL in red wines at 0.05–0.62 µg/L. These amounts could be of physiological interest since the MEL detected in wine was higher than its circulating levels in humans during the day (10 pg/mL) [19]. 

Several factors can affect the concentration of MEL in red wine, including agrochemicals (i.e., plant activators, treatment with copper) and winemaking practices (i.e., barrel-aging, content of sulphur dioxide, and the use of fining agents), fermenting microorganisms (different strains of both *Saccharomyces cerevisiae* and non-*Saccharomyces* yeasts), and maybe even the grape/wine composition (i.e., phenol content, antioxidant capacity) [18,26]. However, further investigations will be carried out in order to elucidate the effects of both winemaking and grape/wine composition. Most of the MISs were detected at trace levels except for MIS 1, for which the concentrations ranged from 0.58 ± 0.03 µg/L to 1.97 ± 0.11 µg/L (Table 4), more than one order of magnitude higher in comparison to MEL. MIS biosynthesis is still not clear, and their antioxidant and cytoprotective activities are dependent on the position of two side chains in the indole ring [40]. 

## 4. Conclusions

The proposed analytical method allowed the detection and reliable quantification of TRP, TEE, and MEL simultaneously. In general, this protocol may represent a useful tool for monitoring the release of MEL and TEE, even when their concentration is very low (<0.02 µg/L), and their fate throughout the wine production and storage. Moreover, the sample preparation method and chromatographic conditions presented in this study allowed the detection of five MISs, for which the molecular structure, in particular the position of two side chains in the indole ring, will be evaluated.

The obtained results suggested the amounts of MEL detected in the wine samples may be of biological importance, as MEL was higher than its circulating levels in humans during the day; bioavailability studies are necessary to confirm this hypothesis. TEE is more highly concentrated than MEL (over one thousand times) in Nebbiolo wine samples, as well as MIS 1, confirming the outcome of previous works [24,26], even though the origin and the putative nutritional role of this molecule have not been elucidated yet. Since chromatographic analysis does not allow getting information about the chemical structure of MISs, further investigations will be carried out in order to clarify the position of two side chains in the indole ring for the MISs detected. We can suppose that the proper choice of the winemaking procedures could lead to higher levels of MEL, TEE, and MISs, and this needs further investigation.

## Figures and Tables

**Table 1:** Linearity obtained with standard synthetic wine solutions by fluorescence (FLD) and mass spectrometry (MSD) detectors.

	FLD			MSD		
Compound	Concentration Range (μg/L)	Linearity		Concentration Range (μg/L)	Linearity	
		Equation	*r*		Equation	*r*
TRP	110–5500	39.03 × *x* − 0.08	0.996	110–5500	3389.30 × *x* + 49.99	1
TEE	50–2000	0.064 × *x* + 7.308	0.998	5–200	23.40 × *x* + 111.85	0.999
MEL	20–500	0.15 × *x* + 0.41	1	1–200	15.04 × *x* + 109.10	0.998

The equations and the correlation coefficients (*r*) were calculated by means of linear regression. *x*, concentration; TRP, tryptophan; TEE, tryptophan ethyl ester; MEL, melatonin.

**Table 2:** Linearity, limits of detection (LOD) and quantification (LOQ), recovery (%), and repeatability (as relative standard deviation, %RSD) for the analytical method developed in HPLC-MS.

Compound	Concentration Range Added (μg/L)	SWS		Spiked Red Wine		LOD (µg/L) (*n* = 3)	LOQ (µg/L) (*n* = 3)	Recovery (%) (*n* = 6)		Repeatability (%RSD) (*n* = 9)	
		Equation	*r*	Equation	*r*			SWS	Spiked Red Wine	SWS	Spiked Red Wine
TRP	110–5500	3284.2 × *x* + 328.3	0.999	3257.4 × *x* + 3132.1	0.995	0.75	1.25	89	84	9.1	10.5
TEE	5–250	659.2 × *x* − 440.4	0.997	677.4 × *x* + 3861.0	0.999	0.038	0.12	88	76	6.5	7.9
MEL	0.05–250	242.2 × *x* + 2748.6	0.996	269.5 × *x* + 840.1	0.997	0.0023	0.018	86	79	4.6	5.4

The equations and the correlation coefficients (*r*) were calculated by means of linear regression. Recovery and repeatability values were determined at two and three different concentration levels, respectively. SWS, synthetic wine solution; *x*, concentration; TRP, tryptophan; TEE, tryptophan ethyl ester; MEL, melatonin.

**Table 3:** Identification of MEL (melatonin), TEE (tryptophan ethyl ester), and MISs (MEL isomers) by HUPLC/ESI-QTRAP.

Compound	Exact Mass	MS/MS Fragmentation	
	[M + H]^+^	MS/MS Fragments	Collision Energy (µV)
MEL	233.1	188.1	30
		216.1	30
		174.1	30
TEE	233.1	174.1	20
		159.0	36
		130.1	55
		178.1	29
MIS 1	233.1	141.0	20
		216.0	20
		174.1	20
MIS 2	233.1	141.1	20
		196.0	20
MIS 3	233.1	130.0	50
MIS 4	233.1	141.0	20
		216.0	20
		174.1	20
MIS 5	233.1	141.0	35
		159.0	35

**Table 4:** Levels of TRP (tryptophan), TEE (tryptophan ethyl ester), MEL (melatonin), and MEL isomers (MISs) in red wine samples. Data are expressed as mean ± standard deviation.

Sample Code	TRP	TEE	MEL	MIS 1	MIS 2	MIS 3	MIS 4	MIS 5
	mg/L	µg/L	µg/L	µg/L	µg/L	µg/L	µg/L	µg/L
Red wine 1	3.85 ± 0.40	172.2 ± 13.6	0.057 ± 0.003	1.64 ± 0.09	<LOQ	<LOQ	0.0043 ± 0.0002	<LOQ
Red wine 2	4.39 ± 0.46	212.0 ± 16.8	0.062 ± 0.003	1.97 ± 0.11	<LOQ	<LOQ	0.0041 ± 0.0002	<LOQ
Red wine 3	1.56 ± 0.16	256.2 ± 20.2	0.063 ± 0.004	0.74 ± 0.04	<LOQ	<LOQ	<LOQ	<LOQ
Red wine 4	1.02 ± 0.11	223.2 ± 17.6	0.038 ± 0.002	0.67 ± 0.04	<LOQ	<LOQ	<LOQ	<LOQ
Red wine 5	0.98 ± 0.10	113.0 ± 8.9	0.046 ± 0.003	0.58 ± 0.03	<LOQ	<LOQ	<LOQ	<LOQ
Red wine 6	0.84 ± 0.09	92.9 ± 7.3	0.054 ± 0.003	0.86 ± 0.05	<LOQ	<LOQ	<LOQ	<LOQ
Red wine 7	0.44 ± 0.05	71.7 ± 5.7	0.063 ± 0.004	0.91 ± 0.04	<LOQ	<LOQ	<LOQ	<LOQ
Red wine 8	0.57 ± 0.06	74.4 ± 5.9	0.038 ± 0.001	0.80 ± 0.04	<LOQ	<LOQ	<LOQ	<LOQ
